# Eco-friendly microencapsulation of *Lacticaseibacillus paracasei* using *Ficus pumila* seed extract: A novel plant-based delivery system enhancing probiotic stability and gastrointestinal tolerance

**DOI:** 10.14202/vetworld.2025.2039-2050

**Published:** 2025-07-27

**Authors:** Watcharapong Mitsuwan, Chonticha Romyasamit, Rungruedee Kimseng, Tanakamol Mahawan, Sasi Vimon

**Affiliations:** 1 Akkhraratchakumari Veterinary College, Walailak University, Nakhon Si Thammarat, 80160, Thailand; 2 One Health Research Center, Walailak University, Nakhon Si Thammarat, 80160, Thailand; 3 Center of Excellence in Innovation of Essential Oil and Bio-active Compound, Walailak University, Nakhon Si Thammarat, 80160, Thailand; 4 School of Allied Health Sciences, Walailak University, Nakhon Si Thammarat, 80160, Thailand; 5 Sanders-Brown Center on Aging, College of Medicine, University of Kentucky, Lexington, KY 40506, USA

**Keywords:** *Ficus pumila*, gastrointestinal stability, *Lacticaseibacillus paracasei*, plant-based matrix, probiotic microencapsulation, storage viability, thermal resistance

## Abstract

**Background and Aim:**

Probiotic viability remains a critical challenge during gastrointestinal (GI) transit, storage, and feed processing. Conventional encapsulation materials often fail under acidic and thermal stress. This study aimed to develop and characterize a novel, eco-friendly microencapsulation system using *Ficus pumila* (FP) seed extract as a natural encapsulating matrix for *Lacticaseibacillus paracasei* (LP) WU2502, enhancing its functional resilience and storage stability.

**Materials and Methods:**

Microcapsules containing LP and FP were formulated through ionic gelation using calcium chloride. Physicochemical properties were assessed using scanning electron microscopy and Fourier-transform infrared (FTIR). Functional evaluations included encapsulation efficiency (EE), swelling kinetics, controlled release in simulated gastric and intestinal fluids, stress tolerance (acid, bile, enzymes, thermal), and viability during 60-day storage at 4°C and 25°C.

**Results:**

The LP/FP microcapsules demonstrated high EE (80.5%) and spherical morphology (~200μm). FTIR confirmed the presence of ionic and hydrogen bonding in the matrix. The system exhibited pH-responsive swelling and controlled release, reaching 89.17% cumulative release in intestinal fluid. Encapsulated cells showed significantly improved tolerance to acidic pH, bile salts, digestive enzymes, and heat compared to free cells (p < 0.05). After 60 days, the viability of encapsulated cells remained above 60% at both storage temperatures, while free cell viability dropped by over 85%.

**Conclusion:**

FP seed extract offers a biodegradable, plant-derived alternative for probiotic encapsulation. The developed LP/FP system effectively enhances probiotic survival under GI and thermal stress and during extended storage. These results support its application as a sustainable delivery platform for animal feed and functional food formulations.

## INTRODUCTION

Probiotics, including lactic acid bacteria, are beneficial microorganisms naturally residing in various systems of both humans and animals, such as the gastrointestinal (GI) tract, reproductive system, and oral cavity [1]. These microbes are widely incorporated into human and animal diets to promote growth, enhance immunity, and support overall health. *Lacticaseibacillus paracasei* (LP) WU2502, previously classified as *Lactobacillus paracasei* [2], was isolated from Palmyra palm sugar and identified by our research group as a promising probiotic candidate. This strain demonstrated the ability to produce antibacterial compounds effec-tive against a range of foodborne and intestinal patho-gens [3]. In addition, LP WU2502 exhibited desirable probiotic traits, including autoaggregation, adhesion to surfaces and Caco-2 cells, and resistance to harsh GI conditions such as low pH, pancreatin, and bile salts [3]. However, the *in vivo* environment within animal GI tracts may differ significantly from *in vitro* conditions. Therefore, enhancing the viability of this strain is essential for its practical use as a feed additive in animal nutrition.

Microencapsulation offers a promising solution by enclosing probiotics within polymer-based microcapsules composed of organic or inorganic materials [4]. These capsules serve as a protective delivery system, minimizing the detrimental effects of GI stresses on probiotic viability [4]. The technique not only enhances probiotic survival during GI transit but also enables targeted delivery to specific sites within the host [5]. Moreover, microencapsulation contributes to the extended shelf-life of probiotic products during storage [6]. Various microencapsulation techniques exist, including freeze-drying [7] and liposome-mediated encapsulation [8]. In a recent study, our team successfully encapsulated *Pediococcus acidilactici* using sodium alginate (SA) and calcium chloride as encapsulating agents [6]. Although SA, a natural polysaccharide derived from brown algae, is widely used, its extraction involves the use of multiple chemical agents, including alkaline solutions, sodium carbonate, and sodium hydroxide (NaOH) [9]. This has led to growing interest in alternative, eco-friendly materials suitable for encapsulating live microbial supplements.

Microencapsulation technologies are increasingly being adopted in animal nutrition to protect sensitive bioactive compounds during feed processing and storage [10]. Given the economic pressures in livestock production, especially concerning feed additives, it is critical to utilize cost-effective and sustainable encapsulating materials [11]. Accordingly, attention has shifted toward natural plant-derived polymers.

Plant-based biopolymers have gained significant interest due to their biodegradability, low toxicity, and compatibility with green chemistry principles. These materials offer a sustainable alternative to synthetic polymers, reducing environmental impact and dependence on chemically intensive processes. One such promising material is *Ficus pumila* (FP) var. *awkeotsang*, commonly known as jelly fig, a native plant of Asia belonging to the Moraceae family. The aqueous extract of FP fruit possesses numerous bioactive properties, including antibacterial, antioxidant, and anti-inflammatory effects [12, 13]. Moreover, non-starch polysaccharides extracted from jelly fig have been reported to exhibit blood glucose-modulating activity in food products [12]. The seeds of FP are traditionally used in dessert preparations due to their unique jelly-forming capacity, attributed to their high pectin content and rich nutritional profile [14]. The viscous pectic component is primarily responsible for the gel-forming characteristics that make FP a compelling candidate for probiotic encapsulation.

While microencapsulation is a widely explored strategy for enhancing probiotic viability during processing, storage, and GI transit, the majority of current systems rely on conventional encapsulants such as SA, gelatin, or synthetic polymers. These materials, although effective, present several limitations, including susceptibility to acid degradation, large pore sizes allowing premature probiotic release, and environmental concerns related to extraction or production processes. Furthermore, although plant-derived biopolymers offer an eco-friendly alternative, their application in probiotic delivery systems remains underutilized. In particular, there is a significant knowledge gap regarding the use of FP seed extract – a pectin-rich, jelly-forming plant product traditionally used in food – in the context of probiotic microencapsulation. To date, no published studies have systematically evaluated the physicochemical properties, GI resilience, or long-term storage performance of probiotic microcapsules using FP extract as the primary matrix. This represents a critical research void, especially for the development of sustainable, cost-effective delivery platforms tailored for animal feed applications under real-world conditions.

This study aims to develop and characterize a novel, eco-friendly microencapsulation system using FP seed extract as a natural encapsulating matrix for LP WU2502. Specifically, we sought to: (1) Fabricate probiotic-loaded microcapsules using ionic gelation; (2) Assess the physicochemical properties of the resulting microcapsules through morphological and structural analysis; (3) Evaluate their performance under simulated GI conditions, including pH-responsive swelling and controlled release; (4) Determine the encapsulated probiotic’s tolerance to acid, bile salts, digestive enzy-mes, and thermal stress; and (5) Investigate the viability of encapsulated cells during long-term storage at refrigerated and ambient temperatures. By addressing these objectives, the study seeks to fill a critical gap in the literature and contribute to the advancement of plant-based probiotic delivery technologies for functional food and livestock applications.

## MATERIALS AND METHODS

### Ethical approval

The study was authorized by the Biosafety Regulation for Scientific Experiments (Ref. No. WU-IBC-66-042) of Walailak University, Nakhon Si Thammarat, Thailand.

### Study period and location

This study was conducted from January 2024 to April 2024. All experiments were carried out at the Laboratory of Bacteriology, Walailak University, Nakhon Si Thammarat, Thailand.

### Probiotic culture and growth conditions

LP WU2502, a probiotic originally isolated from Palmyra palm sugar and characterized as a probiotic candidate by our research group [3], was used in this study. The bacterium was cultured in Mann–Rogosa Sharpe (MRS) broth (HiMedia, India) and MRS agar (HiMedia) and incubated at 37°C for 24 h [15]. The bacterial culture was stored in MRS broth containing 20% glycerol at −80°C until it was used. To prepare the bacterial culture, the bacterium was streaked on MRS agar and incubated at 37°C for 24 h. Then, 3–5 colonies of LP WU2502 were inoculated in 10 mL of MRS broth and incubated at 37°C for 24 h.

### Reagents and materials

All chemicals and reagents used in this study were of analytical grade. SA (Mw = 1.93 × 10^5^ Da, mannuronic/guluronic acid ratio = 1.51), Tween 80 (Sigma-Aldrich, USA), soybean oil, porcine bile extract, pancreatin (from porcine pancreas, containing amylase, lipase, and protease activities), lipase (from porcine pancreas, ≥100 U/mg), pepsin (from porcine gastric mucosa, ≥2,500 U/mg protein), and trypsin (from bovine pancreas, ≥10,000 benzoyl-L-arginine ethyl ester (U/mg protein) were purchased from Sigma-Aldrich, Co. LLC. (3050 Spruce Street, St. Louis, MO 63103, USA). FP seeds were purchased from a local herbal market in Phuket, Thailand. Calcium chloride dihydrate, sodium chloride (NaCl), hydrochloric acid (HCl, 37%), dipotassium hydrogen phosphate (K_2_HPO_4_), NaOH, and trisodium citrate dihydrate were purchased from Merck KGaA (Frankfurter Str. 250, 64293 Darmstadt, Germany). Peptone water was purchased from HiMedia.

### Preparation of FP-based microcapsules

FP seeds (20 g) were homogenized in 100 mL of deionized (DI) water, followed by the addition of 2 mL of Tween 80 and stirring (300 rpm) for 20 min to prepare the extract. Subsequently, 20 mL of the probiotic suspension was added while stirring continuously. The resulting mixture was extruded dropwise through an 18-gauge syringe (needle diameter, 1.8 mm) into a 0.15 mol/L CaCl_2_ solution to induce microcapsule formation, maintaining a 5 cm gap between the syringe and the CaCl_2_ solution. The formed microcapsules, referred to as LP/FP, were collected using a plastic mesh sieve (0.053 mm pore size), washed 3 times with DI water, and dried in a hot-air oven at 50°C overnight.

### Encapsulation yield assessment

The enumeration of viable LP cells was performed in triplicate using the drop plate technique on MRS agar, as described by Gbassi *et al*. [16]. Serial dilutions were prepared by adding 1 mL of the sample to 9 mL of 0.1% (w/v) sterile peptone water, followed by homogenization through stirring for 10 min. Subsequently, 20 μL of each dilution was plated onto sterile Petri dishes and incubated at 37°C for 48 h under anaerobic conditions. Viable cell counts were expressed as colony-forming units per milliliter (CFU/mL). To evaluate the encapsulation efficiency (EE) of LP within the LP/FP microcapsules, 1 g of each microcapsule was suspended and homogenized in 10 mL of sodium citrate solution. The released bacterial cells were enumerated on MRS agar. EE, % was calculated using the following equation [17]:

EE (%) = (N/N_o_) × 100

Here, N is the number of viable entrapped cells released from the microcapsules, and N_o_ is the number of free cells added to the LP/FP microcapsules.

### Scanning electron microscopy (SEM)

The shape and surface morphology of the SA and LP/FP microcapsules were examined using a JEOL SEM-IT300 (SEM-Zeiss, Munich, Germany). To observe the internal structure, the microcapsules were sectioned transversely using a razor blade.

### Fourier transform infrared (FTIR) spectral analysis

The chemical structures of the microcapsules, including SA and LP/FP, were analyzed using a Bruker Alpha FTIR spectrometer (Perkin Elmer [Spectrum One]/Bruker [Tensor 27], Waltham, USA) equipped with an attenuated total reflectance accessory in the spectral range of 4000–400 cm^−1^.

### Swelling kinetics in simulated GI fluids

The swelling behavior of LP/FP microcapsules was evaluated under simulated poultry GI conditions using the methodology described by Azarkhavarani *et al*. [18]. Fresh preparations of simulated gastric fluid (SGF) and simulated intestinal fluid (SIF) were performed following the protocol established by Vimon *et al*. [19]. SGF was prepared by first dissolving 2.0 g of NaCl in approximately 800 mL of distilled water. Subsequently, 3.2 g of porcine pepsin was added to the solution, and the mixture was stirred gently until the solution was completely dissolved. The pH was then carefully adjusted to 1.2 by adding 0.1 N HCl dropwise under constant stirring (approximately 8.5 mL). The final volume was then adjusted to 1L using DI water. SIF was prepared by dissolving 6.8g of K_2_HPO_4_ in 190mL of 0.1M NaOH solution. The pH was adjusted to 7.4, and the final volume was subsequently increased to 1L with DI water.

For the gastric phase simulation, approximately 2.0 g ± 0.05 g of LP/FP microcapsules were immersed in 100 mL of SGF. The pH was sequentially adjusted from 1.2 to 2.5 to reflect the physiological conditions of the poultry proventriculus, which typically exhibits an acidic pH in the range of 2.0–3.0. Incubation was performed at 39.5°C ± 0.5°C using a Memmert WNB 14 thermostatic water bath, and samples were collected at 20-min intervals from 20 to 180 min. At each interval, the microcapsules were filtered and weighed to determine swelling, and the corresponding SGF samples were preserved for the intestinal phase. In the intestinal phase simulation, a mixture comprising trypsin solution (2 mg/mL, 1 mL), bile solution (40 mg/mL, 14 mL), pancreatic solution (3.2 mg/mL, 7.5 mL), and (SIF, 7.5 mL) was added to the SGF samples previously collected by sequentially transferring microcapsule batches from the gastric phase. The pH of the mixture was adjusted sequentially to 5.5 and then to 7.0. Microcapsules were incubated at 39.5°C ± 0.5°C for durations of 220 and 240 min at each pH, respectively. Following incubation, the microcapsules were retrieved and weighed at each specified time point. The swelling percentages were calculated using the following equation [18]:

DS = ([Ws − W_o_]/W_o_) × 100

Where W_o_ and Ws are the weights of the dry and swollen microcapsules, respectively, after 4 h.

### Controlled release studies

The release behaviors of the LP/FP microcapsules were evaluated following the protocol described by Mitsuwan *et al*. [6]. The incubation of microcapsules in SGF and SIF under conditions similar to those used in the swelling studies. At each designated time point, a 1mL aliquot of the supernatant was carefully collected, and the concentration of released bacterial cells was determined using the pour plate method on MRS agar (HiMedia). The cell release index was then calculated according to the specified equation:

Cell release (%) = (B_1_/B_0_) × 100

B_0_ = Bacterial count at the initial phase (log CFU/mL),

B_1_ = Bacterial count released at various time intervals (Log CFU/mL).

### Stress tolerance assays (Acidic, enzymatic, and thermal)

Tolerance characteristics were evaluated in triplicate using 5 mL of free LP cells and 5.0 g ± 0.5 g of LP/FP microcapsules, as described by Mitsuwan *et al*. [6]. Before incubation, free cells and encapsulated probiotics suspended in buffer without treatment were used as control samples for comparative purposes. Acid tolerance was assessed by immersing the samples in 20 mL of 0.2 M citrate-phosphate buffer at pH 2.0. Bile salt tolerance was determined by incubating the samples in a solution prepared by dissolving 3.0 g of porcine bile extract in 100 mL of DI water. Pancreatin tolerance was evaluated by suspending 1.0 g of pancreatin (from bovine pancreas) in 100 mL of DI water containing the sample. All solutions were incubated in a thermostatically controlled water bath with agitation at 39.5°C ± 0.5°C for 30 min. For thermal tolerance assessment, samples were exposed to 85°C for 1 min to simulate feed pelleting conditions. Immediately after each treatment, the viability of the probiotics was determined using previously described methods.

### Storage stability evaluation

The storage stability of free LP and LP/FP microcapsules was evaluated in triplicate following the methodology described by Vimon *et al*. [19]. Briefly, free LP (10 mL) and LP/FP microcapsules (10.0 g ± 0.5 g) were individually sealed in glass vials and wrapped in aluminum foil to minimize exposure to light. The samples were stored at 4°C and 25°C under dark conditions with a relative humidity of 75% for up to 60 days. Subsamples were collected at 30- and 60-day intervals to assess bacterial viability based on tolerance characteristics.

### Statistical analysis

All experiments were performed with three independent biological replicates, each assessed in technical triplicate. Data were analyzed using a one-way analysis of variance to evaluate the influence of acidic conditions, enzymatic activity, and temperat-ure on the viability of free cells and LP/FP microcapsules, as well as the effect of storage conditions on the stability of the probiotic. When significant differences were identified, Tukey’s *post hoc* test was applied for pairwise comparisons among treatments. Results are presented as means ± standard deviation. A significance level of p < 0.05 was used to determine statistical differences among treatments. All statistical analyses were conducted using R software (version 4.3.1; R Foundation for Statistical Computing, Vienna, Austria).

## RESULTS AND DISCUSSION

### Encapsulation yield assessment

**Figure 1:**
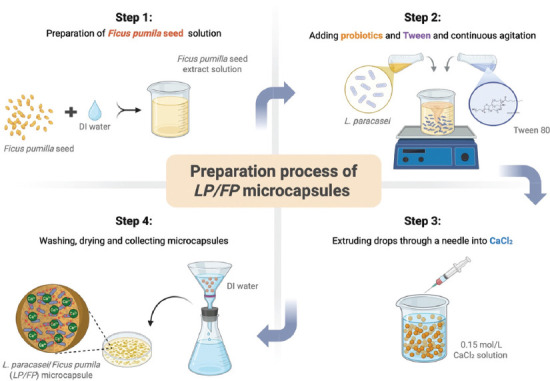
A schematic overview of the preparation of *Lacticaseibacillus paracasei*/*Ficus pumila* microcapsules [Source: Biorender. Com].

A schematic overview of the LP/FP microcapsule preparation process is shown in Figure 1. The EE of the LP/FP microcapsules was determined to be 80.5%, reflecting the successful entrapment of viable LP within the polymeric matrix. This high efficiency was a result of empirical optimization trials conducted before the final experimentation. Key process parameters, including the concentration of FP seed extract, stirring speed, and cross-linking time, were systematically varied and assessed for their impact on encapsulation performance. A stirring speed of 300 rpm was selected to ensure uniform mixing and cell distribution. A cross-linking time of 15 min in a 0.15 M CaCl_2_ solution was found to produce capsules with optimal integrity and minimal cell leakage. The concentration of the FP extract was optimized based on its preliminary gelation ability and polymeric cohesion. The combination of ionic gelation and calcium-mediated crosslinking with pectic substances in FP contributed to a denser and more cohesive matrix, thereby enhancing cell retention and improving encapsulation outcomes.

Comparable encapsulation efficiencies have been reported in previous studies on alginate-based systems. Champagne *et al*. [20] observed EE values ranging from 70% to 85% for various lactic acid bacteria encapsulated in alginate matrices. Similarly, Chen *et al*. [21] achieved encapsulation efficiencies exceeding 80% by optimizing the formulation parameters and using biocompatible encapsulating agents. Thus, the EE obtained in the present study is consistent with, or slightly superior to, values reported in the literature, underscoring the potential of the LP/FP formulation for use in functional food and animal feed applications where microbial stability is critical.

### SEM

**Figure 2:**
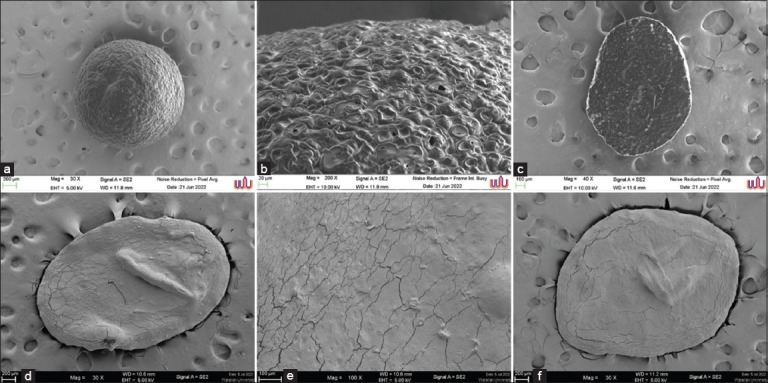
Scanning electron microscopy images. (a) Sodium alginate microcapsule, (b) Surface of (a), (c) Cross section of (a), (d) *Lacticaseibacillus paracasei*/*Ficus pumila* microcapsule, (e) Surface of (d), and (f) Detail on surface of (d).

The surface morphologies of the SA and LP/FP microcapsules were examined using SEM (Figure 2). As shown in Figures 2a and 2b, the SA matrix exhibited a distinct porous network structure with pore diameters ranging from 52 μm to 65 μm (Figure 2c). These porous features are characteristic of ionically cross-linked alginate hydrogels formed through the interaction of divalent calcium ions (Ca^2+^) from CaCl_2_ with the carboxyl groups of the alginate chains. This interaction promotes the formation of the well-known “egg-box” structure, leading to a stable, interconnected three-dimensional network [9].

While the resulting matrix enhances mechanical stability, elasticity, and water retention capacity – properties desirable for probiotic encapsulation – alginate also presents inherent limitations. Notably, alginate gels are sensitive to environmental factors such as ionic strength and pH fluctuations, which can destabilize the cross-linked network and lead to the premature release of encapsulated cells. In addition, the relatively large pore size of alginate matrices can permit the diffusion of small molecules, oxygen, and other harmful agents, reducing the long-term viability of entrapped probiotics [22]. Furthermore, the weak mechanical resistance of alginate microcapsules under acidic conditions, such as those encountered in gastric environments, can compromise their protective capability [23].

To address these limitations, FP extract was investigated. As shown in Figures 2d-f, LP/FP microcapsules exhibit an oval morphology with a slightly flattened structure, a coarse surface texture, and visible surface cracks. Coarse surface refers to the rough texture typical of plant-based hydrogels and is not detrimental, potentially aiding interfacial adhesion. In contrast, “surface cracks” are minor fissures caused by drying but do not compromise EE (80.5%) or probiotic viability. These features are consistent with those observed in previous plant-based encapsulation studies by Champagne *et al*. [20] and Wang *et al*. [24], which may indicate a robust internal structure.

The average diameter of the microcapsules was approximately 200 μm, which falls within the optimal particle size range for inclusion in animal feed formulations. This size facilitates uniform mixing with feed components, minimizing segregation, and ensuring homogeneous distribution throughout the feed matrix [25]. The morphological features observed in the LP/FP microcapsules could be attributed to several contributing factors, including the physicochemical properties of the encapsulating solution, particularly its viscosity and polymer composition, droplet formation technique used during encapsulation, and efficiency of ionic crosslinking between the polymers and calcium ions [24].

The surface roughness and presence of cracks are likely the result of moisture loss during the drying process or structural shrinkage associated with hydrogel crosslinking, a phenomenon frequently reported in plant-based or alginate-derived encapsulation systems [16]. Moreover, the inclusion of FP extract in the matrix may have also influenced the surface morphology by introducing structural heterogeneity, potentially leading to irregularities in the smoothness and compactness of the microcapsules. Similar morphological characteristics have been documented in other microencapsulation studies. Mokarram *et al*. [26] reported rough-surfaced probiotic microcapsules attributed to high crosslinking density and drying conditions. Likewise, Krasaekoopt *et al*. [27] observed that alginate-based beads encapsulating probiotics often develop surface cracks and irregularities when exposed to dehydration or freeze-drying. Although such features may alter surface integrity, they do not necessarily compromise the EE or the protective function of the microcapsule.

### FTIR spectral analysis

**Figure 3:**
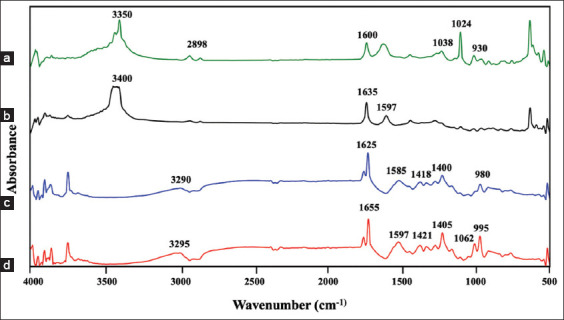
Fourier transform infrared spectra of (a) *Ficus pumila* (FP) seed extract, (b) Free *Lacticaseibacillus paracasei* (LP) WU2502 cells, (c) FP microcapsule without probiotic cells, and (d) LP/FP microcapsule.

FTIR spectroscopy was used to investigate the functional groups and potential chemical interactions involved in the formation of the LP/FP microcapsules (Figure 3). The FTIR spectrum of FP extract (Figure 3a) exhibited characteristic absorption bands indicative of polysaccharide structures. Prominent peaks were observed at 1038 cm^−1^, 1024 cm^−1^, and 930 cm^−1^, corresponding to C–O–C stretching vibrations within the pyranose ring structure. In addition, a band at 2898 cm^−1^ was attributed to C–H stretching vibrations, while a broad absorption near 3400 cm^−1^ and a sharp band at 1600 cm^−1^ were assigned to O–H stretching and asymmetric carboxylate (COO^-^) stretching, respectively, confirming the abundance of hydroxyl and carboxyl groups in the FP polysaccharides [28].

The FTIR spectrum of free LP (Figure 3b) revealed typical protein-related functional groups, with an amide I band at 1635 cm^−1^ corresponding to C=O stretching vibrations of peptide bonds and a band near 1597 cm^−1^, indicative of N–H bending associated with amine groups. These features are consistent with the presence of surface-exposed proteins and amino acid residues in bacterial cells.

In the spectra of the FP-based capsules without bacterial cells (Figure 3c), vibrations of the carboxylate group were observed at 1585 cm^−1^ (asymmetric stretching) and 1400 cm^−1^ (symmetric stretching). Upon the introduction of calcium ions (Ca^2+^) via CaCl_2_ crosslinking, these bands shifted to 1597 cm^−1^ and 1418 cm^−1^, respectively. This shift indicates ionic interactions between Ca ions and the carboxyl groups of the polysaccharides, resulting in the formation of a cross-linked three-dimensional network.

The gelation mechanism follows the classic “egg-box” model, in which divalent calcium ions bridge adjacent carboxyl groups on the polysaccharide chains, thereby stabilizing the matrix and forming a hydrogel structure that can encapsulate and protect probiotic cells [14, 29].

For the LP/FP microcapsules (Figure 3d), further shifts and changes in peak intensity were observed for the functional groups associated with carboxyl (COOH), hydroxyl (OH), and amino (NH_2_) groups. These spectral changes suggest the occurrence of additional electrostatic interactions and hydrogen bonding among LP, FP-derived polysaccharides, and calcium ions, reinforcing the integrity of the encapsulation matrix.

**Figure 4:**
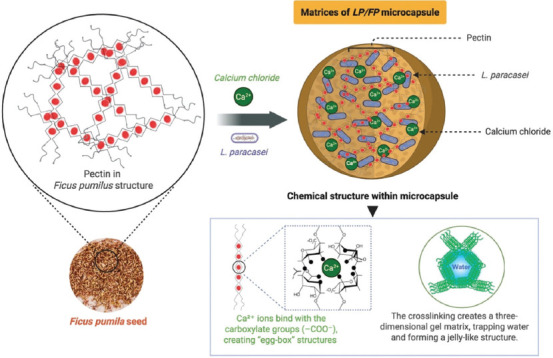
Synergistic interactions between the biopolymers matrices of *Ficus pumila* extract and calcium ions (Ca^2+^) [Source: Biorender. Com].

Collectively, the FTIR findings confirm the success-ful incorporation of LP into the FP matrix, highlighting the pivotal role of calcium-mediated ionic gelation in enhancing the structural stability of the microcapsules (Figure 4). These results are consistent with previous studies by Anal and Singh [30] and Burgain *et al*. [31] on pectin- and alginate-based encapsulation systems, supporting the effectiveness of using natural, plant-derived biopolymers for probiotic delivery applications.

### Swelling behavior and controlled release kinetics

**Figure 5:**
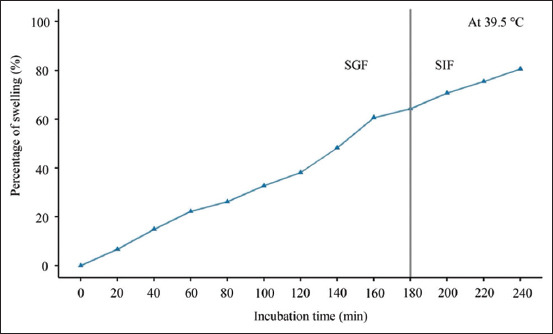
Swelling behavior (%) of *Lacticaseibacillus paracasei*/*Ficus pumila* microcapsules in simulated gastric fluid and simulated intestinal fluid at 39.5°C ± 0.5°C.

To assess the release behavior of LP under simulated GI conditions, swelling and cumulative release studies were conducted in SGF and intestinal fluid (SIF). As illustrated in Figure 5, the swelling ratio of the LP/FP microcapsules increased progressively with increasing incubation time in SGF. At 60, 120, and 180 min, the swelling values were 22.17%, 38.10%, and 64.25%, respectively. This progressive increase indicates a gradual uptake of water and matrix expansion, with the microcapsules reaching approximately twice their initial weight by 180 min.

Following SGF treatment, the microcapsules were transferred to SIF to simulate intestinal conditions. After SIF exposure, the swelling ratio further increased to 80.60%, reflecting the continued hydration and expansion of the matrix in response to the altered pH environment. The observed swelling behavior underscores the pH-sensitive nature of the encapsulating matrix, which includes FP extract. In the acidic environment of SGF (pH 2–3), partial protonation of calcium ions may reduce cross-linking density, allowing for water penetration and gradual swelling. Upon transfer to the more neutral pH of SIF (pH ~ 6–7), which exceeds the pKa of FP (~3.5–4), the deprotonation of carboxyl groups in the extract likely promotes increased electrostatic repulsion and further water absorption.

This pH-responsive swelling is characteristic of anionic hydrogels and has been similarly observed in a previous study by Etchepare *et al*. [23], where the ionization of functional groups led to enhanced fluid uptake under higher pH conditions. These results suggest that the LP/FP matrix responds dynamically to GI pH changes, making it a promising vehicle for targeted probiotic delivery in animal feed systems.

**Figure 6:**
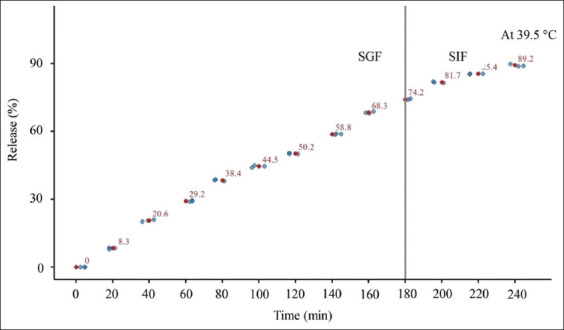
Cumulative releases (%) of *Lacticaseibacillus paracasei*/*Ficus pumila* microcapsules under simulated gastric fluid and simulated intestinal fluid conditions at 39.5°C ± 0.5°C.

The cumulative release profile of LP from LP/FP microcapsules under simulated GI conditions is presented in Figure 6. A clear correlation was observed between the swelling behavior of the microcapsules and the release of viable cells. At 60 min, the release rate reached 29.25%, which is likely attributable to an initial burst effect. This phenomenon may be explained by the presence of loosely bound or surface-adsorbed LP on the outer layer of the FP matrix, which is more susceptible to rapid desorption on hydration in the acidic gastric environment.

As incubation progressed, the cumulative release in SGF increased steadily, reaching 74.22% at 180 min. This controlled release phase is attributed to the gradual swelling of the matrix and the diffusion of entrapped cells from within the hydrogel network. An acidic pH likely facilitates partial deprotonation of surface groups and localized degradation or loosening of the cross-linked network, thereby enhancing the diffusion rate of the encapsulated bacteria.

Upon transfer to the SIF, a further increase in cumulative release was observed, with values rising to 85.42% at 210 min and ultimately reaching 89.17% at 240 min. The enhanced release under intestinal conditions is consistent with the pH-responsive behavior of the FP hydrogel system. At higher pH levels, the ionization of carboxyl groups within the matrix leads to increased electrostatic repulsion, matrix expansion, and porosity, which facilitate the further diffusion of entrapped bacterial cells.

Etchepare *et al*. [23] reported cumulative release rates of 70%–80% for *Lactobacillus* strains encapsulated in alginate beads after 240 min under similar *in vitro* digestion conditions. Similarly, Ding and Shah [32] demonstrated a two-phase release pattern, with an initial burst followed by controlled release, reaching up to 85% for optimized probiotic formulations. The slightly higher release efficiency observed in the present study may be attributed to the synergistic effect of FP extract on matrix hydration, porosity, and biopolymer interactions, which enhance bacterial diffusion.

Overall, the LP/FP microcapsules exhibited controlled and pH-responsive release behaviors, supporting their potential for targeted delivery of probiotics to animal feed applications where GI resilience is essential.

### Stress tolerance assays (acidic, enzymatic, and thermal)

**Figure 7:**
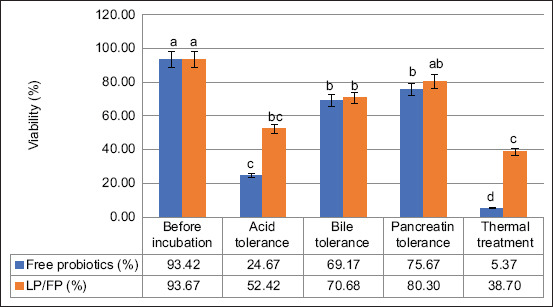
Viability (%) of free cells and *Lacticaseibacillus paracasei*/*Ficus pumila* microcapsules were treated under stimulated gastrointestinal tract fluids and temperature.

During *in vivo* delivery, probiotic bacteria such as LP are exposed to a series of harsh physiological environments, including exposure to gastric acid, bile salts, digestive enzymes, and elevated temperatures during feed processing. To evaluate the protective capability of the FP-based encapsulation system, the viability of free and encapsulated LP was assessed following individual treatments simulating each of these stressors (Figure 7).

Before treatment, both free LP and LP/FP microcapsules exhibited comparable viability, with survival rates of 81.40% and 80.90%, respectively, indicating minimal loss during the encapsulation process. On exposure to acidic conditions (pH 2.0), bile salts, and pancreatin enzyme solution, the viability of LP/FP remained at approximately 50%, demonstrating substantial protection against digestive stress.

In contrast, the viability of free cells decreased significantly across treatments, ranging from 18% to 87% (p< 0.05), depending on the type of stress applied. The enhanced tolerance of the encapsulated cells can be attributed to the structural integrity and chemical characteristics of the FP matrix. The bioactive compounds present in FP, including polyphenols and polysaccharides, contribute to a denser polymeric network, reducing permeability and providing a physical barrier against hydrogen ion penetration and enzymatic degradation [13, 14, 24]. Additionally, the ionic crosslinking between the carboxyl groups in the matrix and calcium ions likely enhances gel rigidity, further contributing to cell protection in acidic and enzymatic environments.

In the case of thermal treatment (85°C for 1 min, simulating feed pelleting conditions), the viability of LP/FP microcapsules was 38.70%, which was significantly higher than that of free LP (5.37%) (p < 0.05). This suggests that the encapsulating matrix offers partial thermal shielding, likely through reduced heat transfer and the protective hydration layer surrounding the microcapsules.

Comparable protective effects have been reported in previous studies. Mokarram *et al*. [26] and Shi *et al*. [33] demonstrated that alginate-based encapsulation improved probiotic survival under acid and bile stress, with viability improvements of 30%–60% compared to free cells. Similarly, Naissinger da Silva *et al*. [34] demonstrated that microencapsulation using natural polymers mitigates heat and GI stress in probiotic systems.

The present study further supports these findings and highlights the unique contribution of FP extract in enhancing matrix density and bio-barrier properties, thereby improving the survival of encapsulated probiotics under multiple stress conditions. These results suggest that LP/FP microcapsules are a promising delivery system for maintaining probiotic viability during GI transit and feed processing, contributing to improved efficacy in functional feed applications.

### Storage stability evaluation

In practical applications, probiotics are often stored for extended periods before being consumed or processed. During storage, environmental factors such as temperature fluctuations, relative humidity, oxygen exposure, and light can significantly affect cell viability by inducing oxidative stress, damaging cell membranes, and compromising metabolic function. These stressors ultimately reduce the efficacy of probiotics and shorten their shelf life.

Therefore, evaluating the stability of probiotic formulations under various storage conditions is essential for ensuring long-term functionality. In the present study, the viability of free LP and encapsulated LP/FP was monitored under two temperature conditions: refrigeration (4°C) and elevated ambient temperature (25°C).

As shown in Table 1, the viability of free LP decreased gradually within the first 30 days of storage and decreased significantly by approximately 85% ± 1.9% after 60 days (p < 0.05). This loss is likely due to progressive degradation of the bacterial cell membrane and cytoplasmic leakage, both of which are intensified by oxidative and thermal stress during storage.

**Table 1:** Comparative viability of *L. paracasei* WU2502 and LP/FP microcapsules as functions of storage temperature and storage duration.

Sample	Storage time (day)	Survival rate at 4°C (%)	Survival rate at 25°C (%)
*L. paracasei* WU2502 (Free cells)	0	82.10^a^ ± 0.11	80.45^a^ ± 0.13
	30	55.15^b^ ± 0.02	35.50^b^ ± 0.06
	60	30.25^c^ ± 0.07	15.05^c^ ± 0.13
LP/FP microcapsule	0	80.55^a^ ± 0.12	81.05^a^ ± 0.16
	30	75.35^ab^ ± 0.08	72.15^ab^ ± 0.03
	60	66.50^b^ ± 0.09	60.20^b^ ± 0.12

^a,b,c^superscript for significant differences (p < 0.05) compared with before storage (day 0). LP=*Lacticaseibacillus paracasei*, FP=*Ficus pumila*

In contrast, the LP/FP microcapsules exhibited substantial stability under both storage temperatures. After 30 days, no significant reduction in viability was observed at either 4°C or 25°C (p < 0.05), indicating that the encapsulation matrix effectively mitigated environmental stress.

Even after 60 days, the encapsulated cells retained 60.3% ± 2.7%–66.2% ± 2.4% viability, with only a moderate decline (34%–40% reduction). These data support the conclusion that the FP-based encapsulation system provides statistically significant protection (p < 0.05) and stability over time, consistent with prior findings in polysaccharide-based microencapsulation studies.

The enhanced stability of LP/FP during storage can be attributed to the structural and biochemical properties of the encapsulation system. The polysaccharide-rich FP extract likely contributes to a tightly packed matrix, minimizing oxygen and moist-ure permeability. In addition, the gel-like environment created by alginate and FP offers mechanical protection, maintains internal moisture balance, and reduces metabolic activity during storage, all of which are crucial for prolonging probiotic viability.

These findings agree with previous studies. Su *et al*. [35] reported improved storage stability of lactic acid bacteria when encapsulated in polysaccharide-based systems, maintaining viability above 60% after 60 days at 25°C. Similarly, Azarkhavarani *et al*. [18] demonstrated that alginate microcapsules can reduce oxidative degradation during storage.

The slightly higher stability observed in the present study may be due to the additional antioxidant components present in FP, which could provide further protection against reactive oxygen species. Overall, our results indicate that the LP/FP encapsulation system offers significant advantages in preserving probiotic viability during storage, making it suitable for incorporation into commercial feed or food products requiring prolonged shelf stability.

## CONCLUSION

This study successfully developed and characterized a novel plant-based probiotic delivery system using FP seed extract to microencapsulate LP WU2502. The EE achieved was 80.5%, reflecting the effective entrapment of viable cells through optimized ionic gelation and calcium crosslinking. SEM imaging revealed a dense, coarse-surfaced microcapsule with a uniform morphology and an average diameter of ~200 μm, making it ideal for inclusion in animal feed. FTIR spectral analysis confirmed the presence of ionic and hydrogen bonding interactions among FP polysaccharides, calcium ions, and probiotic proteins, suggesting strong matrix cohesion.

Functionally, the LP/FP microcapsules demons-trated significant pH-responsive swelling and contr-olled release behavior, with a cumulative release of 89.17% under simulated intestinal conditions. The encapsulated probiotics exhibited superior tolerance to acidic (pH 2.0), enzymatic (bile salts, pancreatin), and thermal (85°C) stress compared to free cells, with post-treatment viabilities of ~50% and 38.7%, respectively. In addition, the LP/FP system showed robust storage stability, maintaining 60.3%–66.2% viability after 60 days at both 4°C and 25°C, while free cell viability declined by ~85%.

The use of FP as a biodegradable, food-grade matrix presents a sustainable and cost-effective alternative to synthetic or chemically extracted encapsulants. This eco-friendly formulation enhances probiotic survival during processing, GI transit, and long-term storage, making it highly applicable in livestock feed and functional food industries.

The study integrates a novel plant-derived material with established microencapsulation techniques, offering a scalable solution with demonstrated sturctural integrity, functional resilience, and thermal protection. The methodology is straightforward, re- producible, and adaptable to other probiotic strains.

The study was conducted under *in vitro* conditions that simulate, but do not fully replicate, the complex dynamics of the animal GI tract. In addition, real-world feed matrix compatibility and sensory or palatability impacts were not assessed.

Further research should include *in vivo* trials in livestock species to validate the probiotic efficacy, gut colonization ability, and health outcomes of the LP/FP system. Exploring the incorporation of prebiotics or bioactive compounds into the matrix could also lead to the creation of a synergistic synbiotic formulation. Optimization for industrial-scale production and feed extrusion compatibility should be addressed for commercial application.

The FP-based microencapsulation system offers a promising, green-technology solution for enhancing probiotic delivery. Its structural and functional advantages support its potential as a reliable platform for improving the efficacy, shelf-life, and stress resilience of probiotics in animal and food biotechnology sectors.

## DATA AVAILABILITY

All the generated data are included in the manuscript.

## AUTHORS’ CONTRIBUTIONS

WM, CR, RK, and SV: Conceived and designed the study. WM and SV: Performed the experiments, analyzed and interpreted the data, and drafted the manuscript. WM, TM, and SV: Statistical analysis and revised the manuscript. All authors have read and approved the final manuscript.
